# How MHCII signaling promotes benign host-microbiota interactions

**DOI:** 10.1371/journal.ppat.1008558

**Published:** 2020-06-29

**Authors:** Mary Melissa Roland, Ahmed Dawood Mohammed, Jason Lee Kubinak

**Affiliations:** University of South Carolina School of Medicine, Department of Pathology, Microbiology, and Immunology, Columbia, South Carolina, United States of America; Geisel School of Medicine at Dartmouth, UNITED STATES

## Introduction: Major histocompatibility complex class II molecules and the microbiota

**Fig 1:**
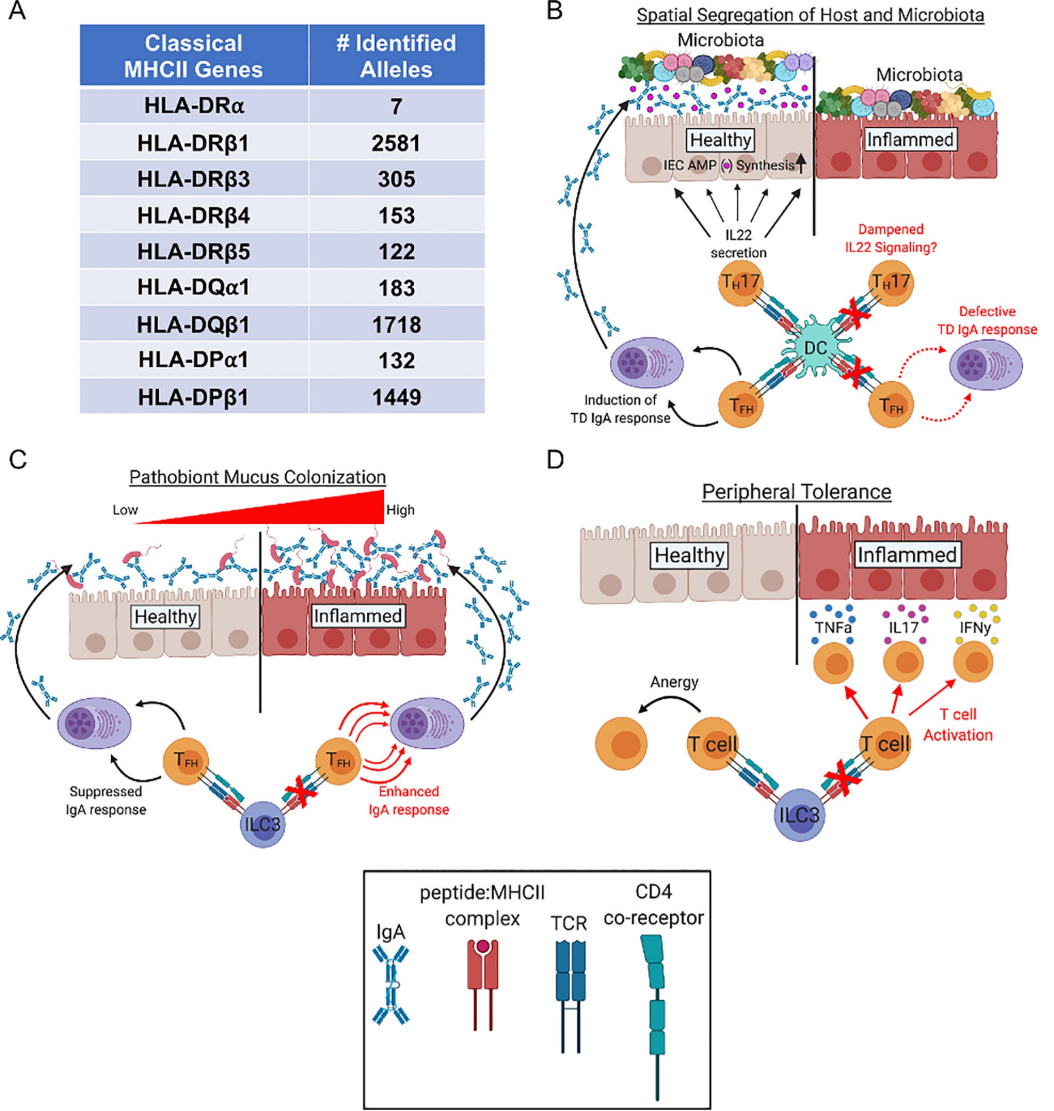
How MHCII can promote benign host-microbiota symbiosis. (A) Classical MHCII genes are “hyper-polymorphic.” The number of identified alleles per each human MHCII gene (termed HLA for “human leukocyte antigen”). Adapted from the work by Robinson and colleagues [1]. (B) MHCII can regulate the spatial segregation between the microbiota and gut epithelium by promoting TD IgA responses in the gut or by regulating IL22 production by TH17 cells. Whether DC-intrinsic MHCII expression promotes IL22 secretion by TH17 (or other) cell types is currently unknown but anticipated. (C) ILC3-intrinsic MHCII expression can promote colonization resistance against pathobionts by limiting TD IgA responses. (D) ILC3-intrinsic MHCII expression can promote peripheral CD4^+^ T cell tolerance against commensal microbes. (B–D) DC- and ILC3-intrinsic MHCII expression have been shown to limit inflammatory gastrointestinal disease in mice. DC, dendritic cell; IFN𝛾, interferon gamma; IgA, Immunoglobulin A; IL22, interleukin 22; ILC3, innate lymphoid cell group 3; MHCII, major histocompatibility complex class II; TD, T cell dependent; TH17, CD4 T helper IL17-producing cell; TFH, CD4+ T follicular helper cell; TNFa, tumor necrosis factor alpha

The major histocompatibility complex (MHC) is a hyper-polymorphic gene-dense region found on Chromosome 6 in humans (the human MHC is termed the HLA for “human leukocyte antigen”). The "hyper"-polymorphic nature of this region stems from the extreme allelic diversity found within classical Class I and class II MHC (MHCII) genes [1] (Fig 1A). MHCII genes encode cell-surface glycoproteins that bind extracellularly derived peptide antigens and present them on the surface of antigen presenting cells (APCs; conventionally, dendritic cells [DCs], macrophages, and B cells). MHCII:peptide complexes engage T cell receptors (TCRs) and CD4 co-receptors which facilitates cognate interactions between CD4^+^ T cells and APCs.

In humans, MHCII deficiency (also known as “bare lymphocyte syndrome type II”) is caused by loss-of-function mutations in genes that drive MHCII surface expression on APCs [2]. The primary immunological features of MHCII deficiency include severe deficits in peripheral CD4^+^ T cells and circulating antibody titers. Severe recurrent infection, especially of the gastrointestinal tract, is the major complication associated with MHCII deficiency in humans. These individuals have an extremely poor prognosis and often succumb to infectious disease in their childhood [3, 4]. Therefore, MHCII-mediated immune responses directed against gastrointestinal microbes is a crucial component of health.

In addition to promoting resistance to pathogenic microbes, MHCII is also emerging as an important pathway regulating interactions between vertebrate hosts and the bacterial community that persistently colonizes the gastrointestinal tract (collectively termed the microbiota). Recent work in MHCII conditional knockout mice has revealed conventional and unconventional roles of MHCII-mediated antigen presentation in promoting benign host-microbiota interactions, and emerging data support that polymorphism at MHCII loci drives variability in microbiota-dependent disease phenotypes.

## How MHCII promotes benign host-microbiota symbiosis

Immunoglobulin A (IgA) is the most abundantly secreted antibody in the gut. T cell dependent (TD) and T cell independent (TiD) B cell maturation contributes to the pool of IgA-secreting plasma cells in the gut. The balance between TiD and TD IgA responses is different between inbred mouse strains suggesting that immunogenetic variation may play an important role in this balance [5]. Using a CD3𝜀^−/−^ adoptive transfer mouse model, Kawamoto and colleagues were the first to explicitly demonstrate that TD IgA responses may have a significant influence on host-microbiota interactions [6]. Specifically, adoptive transfer of FoxP3^+^ regulatory CD4^+^ T (Treg) cells was shown to enhance T follicular helper (T_FH_) cell IL21 secretion in a BCL6-dependent manner. This was associated with increased abundance of IgA^+^ plasma cells in the gut and altered specificity of anti-commensal IgA antibodies, which resulted in significant shifts in microbiota composition. The physiologic impact of this observed effect on microbiota composition was not rigorously addressed in this study, but abnormal shifts in microbiota composition (i.e., dysbiosis) due to IgA deficiency has been associated with enhanced susceptibility to inflammatory gastrointestinal disease in mice and humans [7–9]. MHCII antigen presentation is required for CD4^+^ T cell activation, and recent work in MHCII-conditional knockout mouse models has been instrumental in elucidating the different mechanisms by which MHCII antigen presentation can promote benign host-microbiota interactions by limiting microbiota-dependent inflammatory responses.

Spatial segregation between host tissues and gut microbes is one way that secretory IgA can limit inflammatory responses against intestinal microbes [10, 11]. Conditional deletion of MHCII expression in DCs has been shown to prohibit T_FH_ cell development and abolish anti-commensal TD IgA responses in the gut [12]. Reduced TD IgA responses in this mouse model was shown to result in decreased spatial segregation between the colonic gut epithelium and the microbiota and was associated with the development of a spontaneous microbiota-dependent intestinal inflammatory disease. DC-specific MHCII deletion has also been shown to restrict the development of commensal-specific CD4^+^ T_H_17 cells [13]. T_H_17 cells are important producers of interleukin 22 (IL22), a cytokine that induces antimicrobial peptide secretion by intestinal epithelial cells (IECs), which also promotes spatial segregation between the gut epithelium and the microbiota [14, 15]. IL22 also upregulates the expression of the polymeric Ig receptor (PIgR) [16], which facilitates basolateral transport of IgA to the apical surface of IECs (Fig 1B).

Group 3 innate lymphoid cells (ILCs) are a subset of unconventional CD4^+^CD3^−^ lymphocytes that are enriched in mucosal sites, and a recent study has shown that ILC3s utilize MHCII-mediated antigen presentation to suppress anti-commensal IgA responses in the gut [17]. In this study, it was shown that ILC3s dampen anti-commensal TD IgA responses (by specifically suppressing CD4^+^ T_FH_ cell development) in an MHCII-dependent manner. Absence of ILC3-intrinsic MHCII expression significantly enhanced anti-commensal IgA responses in this study. Surprisingly, increased IgA-coating of gut bacteria enhanced susceptibility to *Citrobacter rodentium*-induced colitis, putatively by enhancing IgA-coating and mucus-colonization by this pathobiont (Fig 1C).

ILC3s have also been shown to promote peripheral tolerance to microbiota-derived antigens in an MHCII-dependent manner. Hepworth and colleagues demonstrated that deletion of MHCII on ILC3s resulted in low-grade systemic inflammation that was associated with CD4^+^ T cell activation [18]. These animals were also shown to develop a spontaneous inflammatory bowel disease (IBD) associated with increased abundance of CD4^+^ T cells expressing the pro-inflammatory cytokines IL17, interferon gamma (IFN𝛾), and tumor necrosis factor alpha (TNF⍺) in the colonic lamina propria (Fig 1D). Adoptive transfer of CD4^+^ T cells from mice whose ILC3s did not express MHCII (but not CD4^+^ T cells from wild type (WT) mice with intact ILC3-intrinsic MHCII expression) were able to drive the development of gastrointestinal disease in RAG1^−/−^ mice but not germfree RAG1^−/−^ mice, indicating that aberrant CD4^+^ T cell activation due to MHCII-deficiency in ILC3s was a microbiota-dependent phenomenon. In humans, specific MHCII alleles are known genetic risk factors for the development of IBDs [19], and aberrant T cell activation is a major driver of IBDs [20].

## Microbiota-dependent phenotypes driven by MHCII polymorphism

Multiple studies in fish, mice, rats, cows, birds, and humans have associated MHCII polymorphisms with shifts in gut microbiota composition [21–30]. MHCII polymorphisms can putatively influence microbiota composition by differentially regulating any of the mechanisms described above. Three independent studies using MHC congenic or coisogenic mouse strains support that MHCII polymorphisms influences host-microbiota interactions by influencing the magnitude, quality, and specificity of anti-commensal IgA responses [21, 22, 30]. Importantly, two of these mouse studies have linked observed MHCII-mediated shifts in microbiota composition to variability in disease susceptibility. For example, Kubinak and colleagues demonstrated that MHCII-mediated differences in microbiota composition could explain patterns of colonization resistance against the enteric pathogen *Salmonella e*. *typhimurium* [21]. In a subsequent study, Silverman and colleagues were able to demonstrate that transfer of microbiota from a mouse strain expressing a diabetes-resistant MHCII allele was able to limit disease progression in a diabetes-prone coisogenic strain [22]. These two studies provide support for the idea that allelic diversity at MHCII loci drives variability in microbiota-dependent disease phenotypes.

## Concluding remarks

Mucosal IgA deficiency may promote the development of gastrointestinal inflammatory disease in humans [8, 31], and specific MHCII alleles have been identified as candidate genetic risk factors for the development of this form of immunodeficiency [32–34]. Collectively, the available evidence to date supports the conclusion that the primary mechanism by which MHCII regulates host-microbiota interactions is by regulating TD anti-commensal IgA responses. We have focused this review on the role of hyper-polymorphic MHCII molecules in the regulation of host-microbiota interactions because variation in the ability to manage this interaction is emerging as a major contributing factor in the pathogenesis of multiple diseases. MHCII molecules play a central role in CD4^+^ T cell activation and the ensuing adaptive immune response against extracellular microbes. Thus, variability in the immune response caused by genetic variation at MHCII loci may be one of the most important underlying genetic factors controlling microbiota-dependent disease phenotypes. This is an important observation for two reasons. First, it suggests that MHCII alleles may be useful as predictive biomarkers of such diseases. Second, it gives us a new perspective on MHC-mediated disease pathogenesis by suggesting that MHC-disease associations can arise as a consequence of how host MHC genotype influences the physiological outcomes of host-microbiota interactions. This perspective may lead to the development of novel therapies or strategies for the treatment of disease. Polymorphism is found within both the promoter regions and exons encoding the peptide-binding grooves of MHCII molecules. Theoretically, both the relative expression of MHCII molecules on APCs and the unique suite of peptide antigens bound by different MHCII molecules can contribute to variability in anti-commensal IgA responses. Currently, the role of MHCII promoter polymorphisms have not been studied in the context of host-microbiota interactions. Finally, it is important to acknowledge that nonpolymorphic MHC molecules (e.g., H2M3 and MR1, respectively) are also emerging as important factors regulating host-microbiota symbiosis [35, 36] but are beyond the scope of this review. Given the tremendous interest in MHC biology by ecologists, evolutionary biologists, and immunologists alike, we look forward to the many exciting discoveries to be made in these important pathways of symbiosis in the years to come.
